# Inhibitory Effect of L-Methionine on *Alternaria alternata* Based on Metabolomics Analysis

**DOI:** 10.3390/jof10020151

**Published:** 2024-02-13

**Authors:** Xianran Zhu, Shaoying Zhang, Youwei Yu, Shengwang Li, Chao Yang, Yuan Chang

**Affiliations:** College of Food Science, Shanxi Normal University, Taiyuan 030000, China; zzz33499571@163.com (X.Z.); yywsxnu@163.com (Y.Y.); liswang98@163.com (S.L.); ych112310@163.com (C.Y.); 16635711152@163.com (Y.C.)

**Keywords:** methionine, *Alternaria alternata*, metabolomics, antifungal mechanism

## Abstract

*Alternaria alternata* is the main pathogenic fungus of postharvest black spots in fruits and vegetables. This study aimed to explore the antifungal activity of methionine on *A. alternata* in vitro and to reveal related antifungal mechanisms through a metabolomics analysis. The results showed that the inhibitory effects of L-methionine (Met) treatment on mycelium growth, spore germination, and the germ tube elongation of *A. alternata* were enhanced with an increase in the Met concentration, but the inhibitory effects decreased when the Met concentration was higher than 50 mmolL^−1^. The results of propidium iodide staining and scanning electron microscopy showed that the Met treatment damaged the plasma membrane integrity of the *A. alternata* spores and caused an irreversible deformation of mycelium. In addition, after the Met treatment, the leakage of electrolytes, nucleic acid, and proteins in the *A. alternata* cells was significantly higher than that in the control group, indicating that the Met treatment increased the permeability of the cell membranes. Eighty-one different metabolites, divided into seven categories, were identified through the metabolomics analysis, including forty-three downregulated metabolites and thirty-eight upregulated metabolites. Among them, these differential metabolites were mainly involved in amino acid synthesis and metabolism, the pentose phosphate pathway, and the TCA cycle. Therefore, the antifungal effect of the Met treatment on *A. alternata* was mainly to damage the integrity of the cell membranes, make nucleic acid and protein contents leak, and affect the TCA cycle, carbohydrate metabolism, amino acid synthesis metabolism, and the metabolic pathways associated with cell membrane biosynthesis. Thus, the growth and development of *A. alternata* were inhibited. The research enriched the investigation of the effect of the antifungal mechanism of Met treatment on *A. alternata* and provided a theoretical basis for the application of Met to prevent and treat postharvest black spots in fruits and vegetables.

## 1. Introduction

Black spot disease is one of the main postharvest diseases of winter jujube fruits, caused by pathogenic microorganisms. *A. alternata*, being the primary pathogen responsible for this disease, exhibits a wide distribution in nature and possesses adaptability [1]. It can infect fruits and vegetables and quickly cause black rot during storage and transportation, such as jujube, blueberry, apple, yellow peach, and so on [2,3,4,5]. In addition, *A. alternata* can produce mycotoxins such as alternarol, alternarol-monomethyl ether, and alkenes [6]. Some of these mycotoxins have acute toxicity, cytotoxicity, genotoxicity, and carcinogenicity, making them harmful to human health [7,8,9]. Traditional chemical fungicides have good effects on inhibiting the infection of *A. alternata* in postharvest fruits and vegetables [10]. However, more and more consumers have begun to pay attention to food safety, which limits the wide application of fungicides [11].

L-methionine (Met), an essential amino acid, acts as an endogenous antioxidant in cells and resists oxidative stress [12]. Additionally, Met can serve as an effective exogenous treatment for enhancing plant resistance against biological or abiotic stresses. For example, Met can effectively suppress grape downy mildew by stimulating the production of H_2_O_2_ and upregulating the expression of defense-related genes [13]. After a mixture of Met and riboflavin was sprayed on a cucumber leaf surface, the activities of antioxidant enzymes such as POD and SOD were significantly improved, and the infection of powdery mildew was significantly reduced [14]. Spraying Met on the leaves of cauliflower increased the accumulation of soluble sugar and reduced sugar, improving the growth ability of cauliflower under drought stress [15]. According to the Food and Drug Administration, Met is one of nine essential amino acids in humans, and it can be safely added to food except for infant foods. Besides being a building block of proteins, Met has also been revealed to regulate metabolic processes, immunity, and enteric function in mammals [16,17]. 

In recent years, metabolomics analysis has been widely used in the study of postharvest antiseptic mechanisms in fruits and vegetables. For example, myrcene stimulated the spore germination of *Penicillium digitatum* by upregulating the content of metabolites involved in glycolysis, the pentose phosphate pathway, and the citric acid cycle [18]. Tea tree oil inhibited the spore germination and mycelial growth of Botrytis cinerea by inhibiting the metabolisms of pyruvate and amino acids [19]. γ-aminobutyric acid destroyed cell structure and reduced the activity of cell-wall-degrading enzymes by downregulating genes related to spore germination and toxin synthesis, inhibiting the hyphal growth of *A. alternata* [20]. However, the inhibitory mechanism of Met against *A. alternata* has not been reported so far.

In this study, the antifungal effect was preliminarily explored by measuring the plasma membrane integrity, membrane permeability, and leakage of the cellular contents of *A. alternata* treated with Met. Afterward, non-target metabolomics was employed to analyze the changes in the related metabolites and metabolic pathways of the treated *A. alternata*. Thus, a possible inhibitory mechanism in the metabolic regulation of Met treatment on *A. alternata* was investigated as well. This research might provide relevant references for the application of Met to control the black spots caused by *A. alternata* in postharvest fruits.

## 2. Materials and Methods

### 2.1. Strain and Chemicals

*A. alternata* (BNCC115062) was purchased from BeNa Culture Collection (Beijing, China). Met (≥99%) was acquired from Shanghai Aladdin Bio-Chem Technology Co., Ltd (Shanghai, China).

### 2.2. Preparation of Spore Suspension

*A. alternata* was cultured in potato dextrose agar (PDA, Qingdao Hope Bio-Technology Co., Ltd., Qingdao, China) at 28 °C for two weeks. Afterward, the plates were flooded with sterile distilled water and then gently rubbed with a sterile glass spreading rod to release spores. Spore suspensions were filtered through four layers of sterile cheesecloth to remove mycelial fragments, and the spore concentration was adjusted to 1 × 10^6^ spores mL^−1^ with the aid of a hemocytometer prior to use.

### 2.3. A. alternata Microbial Index Determination

#### 2.3.1. Determination of the Mycelial Growth Inhibition Rate of *A. alternata*

The effects of Met on the mycelial growth of *A. alternata* were evaluated according to the methods previously reported by Ji et al. [21]. Met was dissolved into a sterilized PDA medium and prepared in concentrations of 0, 5, 25, 50, and 100 mmolL^−1^, respectively. The PDA medium was poured into a petri dish (90 mm in diameter). A 12 mm plug of mycelial agar, obtained from the edge of 3 d old cultures of *A. alternata*, was transferred in the center of each Petri dish. The plates were cultured for 120 h at 28 °C. The diameter (mm) of the colony zone was determined with a caliper every 24 h. The diameter was measured by the cross method, and the inhibition rate of mycelia growth was calculated according to the following formula.

$$\mathrm{Inhibition}\;\mathrm{rate}\left(\%\right)=\frac{\mathrm{Diameter}\;\mathrm{of}\;\mathrm{control}\;\mathrm{colony}-\mathrm{Diameter}\;\mathrm{of}\;\mathrm{Treated}\;\mathrm{group}\;\mathrm{colony}}{\mathrm{Diameter}\;\mathrm{of}\;\mathrm{control}\;\mathrm{colony}}\times 100$$

#### 2.3.2. Assay for the Spore Germination of *A. alternata*

The spore germination experiment was conducted based on the modified protocol developed by Cheng et al. [22]. In detail, Met was added into the sterilized PDA medium to generate final concentrations of 0, 5, 25, 50, and 100 mmolL^−1^. Afterward, the PDA mediums were poured into Petri dishes. Then, 50 μL of spore suspension with a concentration of 1 × 10^6^ spores mL^−1^ was added and spread with a glass spreading rod evenly. After incubation at 28 °C for 4, 6, 8, 10, and 12 h, the spores were observed through a microscope, and each replicate was counted to be approximately 200 spores, respectively. A spore was considered germinated when the germ tube had a greater or equal length than the spore diameter. The spore germination rate was expressed as the percentage of spore germination to the total spore number.

$$\mathrm{Spores}\;\mathrm{germination}\;\mathrm{rate}\left(\%\right)=\frac{\mathrm{Germinated}\;\mathrm{spores}}{\mathrm{The}\;\mathrm{total}\;\mathrm{spores}}\times 100$$

#### 2.3.3. Determination of the Mycelial Morphology of *A. alternata*

Samples of *A. alternata* were prepared using the modified method described by Li et al. [23]. For scanning electron microscope (SEM) observations, the mycelial cells were fixed with 2.5% (*v*/*v*) glutaraldehyde at 4 °C for 24 h. The mycelial cells were then washed three times with phosphate buffer and post-fixed with 1% osmium tetroxide for 2 h. After being washed three times with the same buffer, the specimens were dehydrated once with 30%, 50%, 70%, 85%, and 95% ethanol and twice with 100% anhydrous ethanol. Each time lasted 15 min. The anhydrous ethanol was replaced by 50% and 100% isoamyl acetate for 20 min each time. In each step, the cells were centrifuged at 8000 rpm and 4 °C for 5 min. Finally, the samples were dried in a freeze/vacuum-drying oven, coated with gold, and examined using a scanning electron microscope (JSM-IT800).

#### 2.3.4. Assay of Plasma Membrane Integrity

The membrane integrity of spores exposed to Met was assessed as described by Zhang et al., with slight modifications [24]. An aliquot of *A. alternata* spore suspension (100 μL, 1 × 10^6^ spores mL^−1^) was incubated with 5 mL of normal saline containing different concentrations of Met. Normal saline was used as the control group. Afterward, the samples were incubated on a rotary shaker (120 rpm/min, 28 °C) for 6 h and then centrifuged (5000 r, 4 °C) for 5 min. The precipitate was washed thrice using normal saline to remove the residual dye, added to 0.1 mL of PI dye (1 mg mL^−1^), and shaken well. It was incubated at room temperature in the dark for 20 min. The samples were observed using a fluorescence microscope.

#### 2.3.5. Determination of Electrolyte Leakage and Cellular Content Leakage

Electrolyte leakage and cellular content leakage were measured according to Ma et al. [25]. The electrolyte leakage from the mycelia was evaluated by measuring the electrical conductivity of the incubation medium with a conductivity meter. *A. alternata* was cultured in PDB at 28 °C and 120 rpm for 2 d. After rinsing, the mycelia were resuspended in 5 mL of sterile deionized water in the presence of Met (0, 5, 25, 50, and 100 mmolL^−1^) and further incubated for another 6 h under the same conditions. The electrical conductivity of the mycelium culture medium was determined after 0, 1, 2, 3, 4, 5, and 6 h, respectively. The change rate of electrolyte leakage was calculated according to the following formula.

$$\mathrm{Change}\;\mathrm{rate}\;\mathrm{of}\;\mathrm{electrolyte}\;\mathrm{leakage}\left(\%\right)=\frac{\mathrm{Conductivity}-\mathrm{Initial}\;\mathrm{conductivity}}{\mathrm{Initial}\;\mathrm{conductivity}}\times 100$$

Subsequently, the filtrates after removing mycelia were used for measuring nucleic acids and protein contents. Ultraviolet spectrophotometry was performed to quantitate nucleic acids and proteins, which were, respectively, quantitated by determining the absorbance at 260 nm (OD_260_) and 280 nm (OD_280_).

### 2.4. Metabolomics Analysis

*A. alternata* strains germinating with and without 50 mmolL^−1^ of Met for 48 h were used for the metabolomics analysis. A total of three samples were collected from each group. The extraction and identification of metabolites and data processing were executed by Nanjing Paisenuo Gene Technology Co., LTD (Nangjing, China), and metabolites were determined by liquid chromatography–mass spectrometry (LC-MS). These metabolic data were processed using MetaboAnalyst 4.0 online software (https://www.metaboanalyst.ca, accessed on 20 March 2023). After automatic scaling of the original data, hierarchical cluster analysis (HCA), principal component analysis (PCA), and orthogonal partial least-squares discriminant analysis (OPLS-DA) were performed. The pathway analysis and mapping of identified metabolites were based on the Kyoto Encyclopedia of Genes and Genomes (KEGG) online database (https://www.genome.jp/kegg/, accessed on 22 March 2023). The significance level of metabolite molecules was set at *p* < 0.05 and VIP > 1 [18].

### 2.5. Data Analysis

All of the tests were performed in triplicate. Data were processed using the software IBM SPSS Statistics Version 25.0 (IBM). The data were expressed as mean ± standard deviation (SD) by measuring three independent replicates and analyzed with a least significant difference test of variance (ANOVA). A value of *p* < 0.05 was considered statistically significant. Graphs were plotted using Origin 2022.

## 3. Results

### 3.1. Effect of Met on the Mycelial Growth of A. alternata

**Figure 1:**
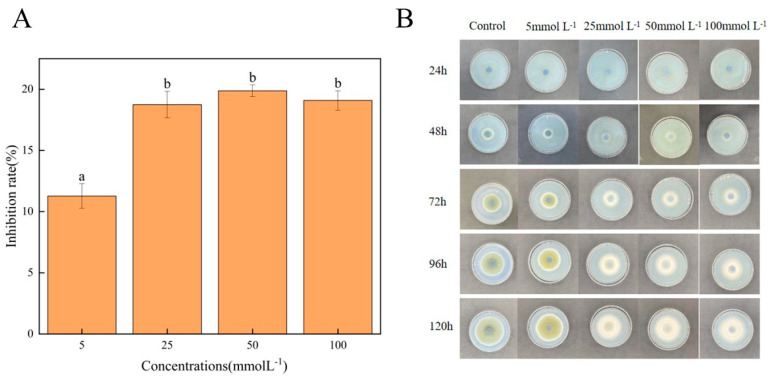
Effect of Met on mycelium growth of *A. alternata*. (**A**) Inhibition rate of mycelial growth after Met treatment for 120 h. Different lowercase letters indicate significant differences between different concentrations at the same time according to Duncan’s multiple range test (*p* < 0.05). All results are the means ± SD. (**B**) Colony morphology of *A. alternata* on PDA plate.

The effects of different Met concentrations on the mycelial growth of *A. alternata* are shown in Table 1. It can be found that the colony diameter increased with the extension of culture time. Met inhibited the mycelial growth of *A. alternata*, and the inhibitory effect first increased and then decreased with increasing Met concentration. The inhibition rate of the group treated with 50 mmolL^−1^ of Met was 18.74% higher than that of the control group after 120 h of culture (Figure 1A). As shown in Figure 1B, with increasing Met concentration, the mycelia became shorter, less easy to pick up, and white in color, and the spore-producing capacity of the mycelium was decreased as well. It was speculated that Met might inhibit the ability of *A. alternata* to produce melanin and spores. The results showed that the inhibitory effects of Met against *A. alternata* were obviously dose-dependent when the treatment concentration was less than 50 mmolL^−1^. With a further increase in Met concentration, the antifungal effect of *A. alternata* decreased. In terms of mycelium growth, 50 mmolL^−1^ of Met showed the best inhibition effect.

### 3.2. Effects of Met on the Spore Germination of A. alternata

**Figure 2:**
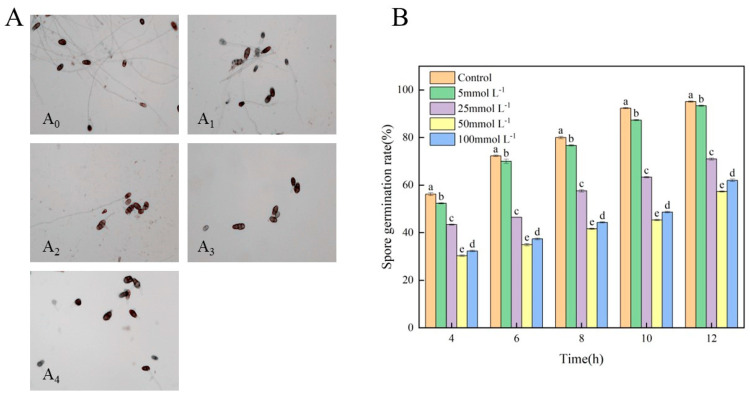
Effect of Met treatment on spore germination of *A. alternata*. (**A**) *A. alternata* spores treated with different concentrations of Met for 12 h (40 × 10): (**A_0_**) Control, (**A_1_**) 5 mmolL^−1^, (**A_2_**) 25 mmolL^−1^, (**A_3_**) 50 mmolL^−1^, and (**A_4_**) 100 mmolL^−1^. (**B**) Spore germination rate. Different lowercase letters indicate significant differences between different concentrations at the same time according to Duncan’s multiple range test (*p* < 0.05). All results are the means ± SD.

The results of the microscopic observation of spore germination are shown in Figure 2A. In the control group, after 12 h of incubation, almost all of the *A. alternata* spores germinated. It was observed that the spores strongly germinated, and the germ tubes were complicated. With an increasing Met concentration, the germination number of spores decreased, and the germ tubes became shorter. The spore growth in the group treated with 100 mmolL^−1^ of Met was similar to that in the group treated with 50 mmolL^−1^ of Met. The spore germination rate of the control group and treatment groups increased with the extension of culture time (Figure 2B). Compared with the control group, the spore germination rate of the groups treated with 25, 50, and 100 mmolL^−1^ of Met decreased by 35.67%, 41.79%, and 33.12% after 12 h, respectively. These results indicate that Met had an inhibitory effect on the spore germination and germ tube elongation of *A. alternata*. The inhibitory effect first increased and then decreased with increasing Met concentration. The concentration of 50 mmolL^−1^ of Met exhibited the strongest inhibitory effect.

### 3.3. Effects of Met on the Mycelial Morphology and Plasma Membrane Integrity of A. alternata

**Figure 3:**
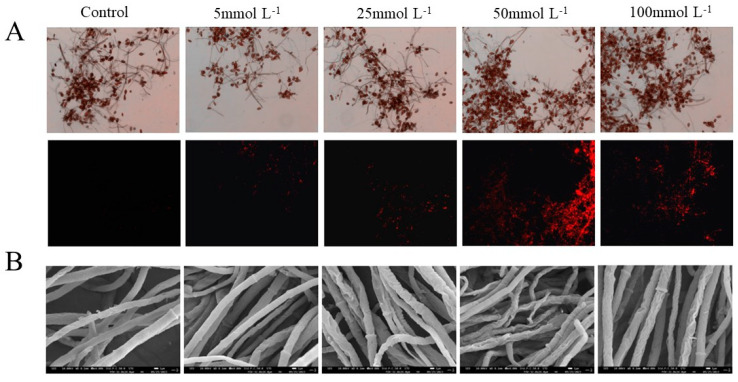
Effects of Met on (**A**) the integrity of spore plasma membranes (40 × 10) and (**B**) the mycelial morphology of *A. alternata* (4000×).

PI dye cannot penetrate the cell membrane of living cells. However, it may cross damaged cell membranes and enter cells, specifically binding to DNA and fluorescing red at a 600 nm wavelength. In Figure 3A, the spores of the control group did not exhibit red fluorescence under excitation wavelength irradiation. Red fluorescence in the groups treated with Met began to appear, indicating that the cell membranes were damaged. Among them, the red fluorescence of the group treated with 50 mmolL^−1^ of Met was the strongest, suggesting that this concentration caused the most serious damage to the cell membranes. The surface morphology of a mycelium reflects whether it grows normally. As shown in Figure 3B, the mycelium morphology of the control group was normal, as the mycelium was linear and the surface was smooth and full. Whereas, the mycelium treated with Met showed abnormal morphology such as depression, distortion, and folding. Especially, 50 mmolL^−1^ of Met showed the most obvious effect on the mycelium morphology of *A. alternata*.

### 3.4. Effects of Met on the Electrolyte and Cellular Leakage of A. alternata

**Figure 4:**
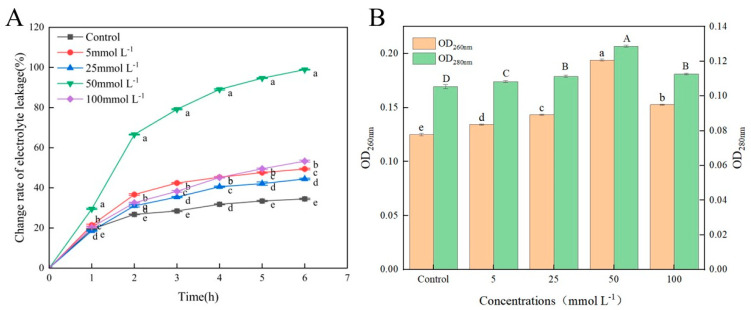
(**A**) Change rate of electrolyte leakage. Different letters mean significant differences according to Duncan’s multiple range test (*p* < 0.05). (**B**) The leakage of nucleic acids and proteins in *A. alternata*. Different uppercase letters indicate a significant difference in OD_280nm_ at the same time, and different lowercase letters indicate a significant difference in OD_260nm_ at the same time (*p* < 0.05). All results are the means ± SD.

Membrane permeability increase and electrolyte exosmosis acceleration may manifest in damage to cell membranes. The effect of the Met treatment on membrane damage was investigated by measuring the changes in electrolyte leakage and cellular leakage. As shown in Figure 4A, electrolyte leakage gradually increased with time. The change rate of electrolyte leakage in the control group increased by 15.11% in 6 h, showing a relatively gentle increasing trend in general. Compared with the control group, the Met treatment could accelerate electrolyte leakage in *A. alternata*. The change rate of electrolyte leakage in the group treated with 50 mmolL^−1^ of Met increased by 69.4% in 6 h, which was the most rapid increase. This indicated that the mycelium of *A. alternata* treated with 50 mmolL^−1^ of Met had the highest membrane permeability and the most serious damage.

Proteins and nucleic acids were the main targets of the mycelial lysate determination, and the change in OD_280nm_ reflects the dissolution of the proteins at different concentrations of Met treatment, while OD_260nm_ indicates nucleic acid leakage. As shown in Figure 4B, nucleic acid and protein dissolution first increased and then decreased with the increase in Met concentration. The absorbance values of the group treated with 50 mmolL^−1^ of Met at 260 nm and 280 nm were 56% and 22.86% higher than those of the control group, respectively. Therefore, after treatment with 50 mmolL^−1^ of Met, the leakage of nucleic acids and proteins in the *A. alternata* mycelium was the most serious.

### 3.5. Metabolomics Data Analysis

#### 3.5.1. PCA Analysis and OPLS-DA Analysis

**Figure 5:**
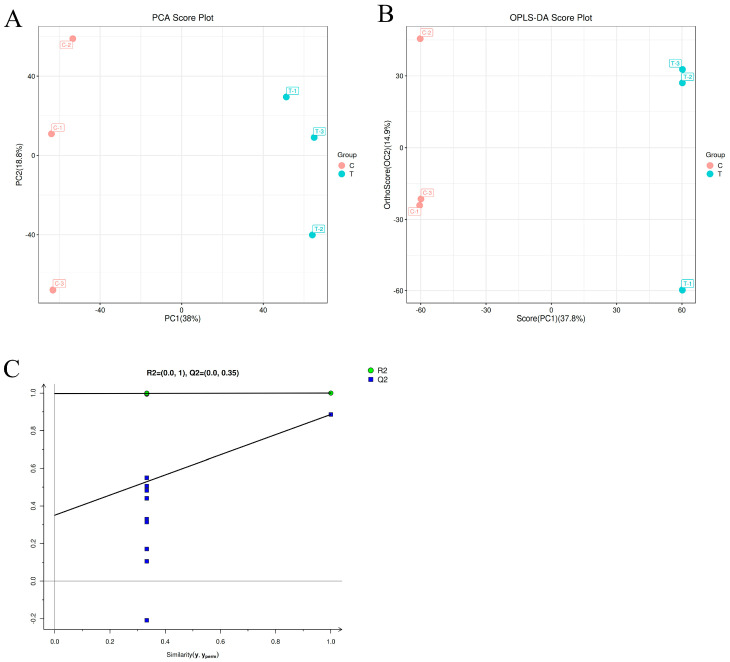
Multivariate data analysis of *A. alternata* metabolites: (**A**) PCA score plot, (**B**) OPLS-DA score plot, and (**C**) OPLS-DA permutation test diagram.

A principal component analysis (PCA) was performed on the metabolites detected in the *A. alternata* mycelium of the control group (C) and the *A. alternata* mycelium of the group treated with 50 mmolL^−1^ of Met treatment (T), respectively. The results are shown in Figure 5A. The contribution rate of PC1 was 38%, and the contribution rate of PC2 was 18.8%. The sum of the contribution rates of the two principal components was 56.8%, which indicated that the two principal components could reflect the main characteristic information of the samples. The two groups of samples were clearly divided into two regions without overlapping, and there were significant differences in the metabolites between them.

Though PCA can effectively extract the main information, it is not sensitive to the variables with little correlation. To solve this problem, the differences between the groups were highlighted to facilitate the subsequent search for different components. An orthogonal partial least-squares discriminant analysis (OPLS-DA) was used to analyze the data (Figure 5B). The contribution rates of PC1 and PC2 obtained by the OPLS-DA analysis were 37.8% and 14.9%, respectively. All the samples from both groups were within the confidence interval, and the pairings were differentiated. The results indicated a significant difference between them, following the PCA findings. In addition, all the blue Q2 points (predictability of model) in Figure 5C are lower than the original blue Q2 points on the far right, indicating that no overfitting took place in the evaluation model and manifesting that the OPLS-DA model was stable and reliable. Therefore, differential metabolites could be screened according to the variable importance projection (VIP) analysis.

#### 3.5.2. Differential Metabolite Screening and Cluster Analysis

**Figure 6:**
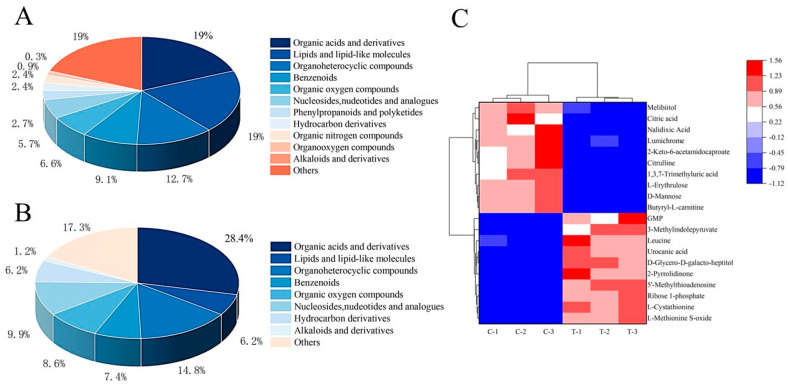
Metabolite profile analysis of *A. alternata*. (**A**) Classification of metabolites identified after treating *A. alternata* with Met. (**B**) Classification of 81 differential metabolites screened after treating *A. alternata* with Met. (**C**) HCA heat map of top 20 differential metabolites.

Differential metabolites were found in group T and group C at a *p* value < 0.05 and VIP > 1. A total of 10,556 metabolites were identified, including 1916 differential metabolites (Table S1), of which 930 were upregulated and 986 were downregulated (Figure S1). Based on the OPLS-DA analysis and differential metabolites screening criteria, a total of 332 metabolites were screened (Table S2). The three categories with the largest proportions of these metabolites were organic acids and derivatives, lipids and lipid molecules, and heterocyclic compounds, accounting for 18.98%, 18.98%, and 12.65% of metabolites, respectively (Figure 6A). Finally, a total of 81 differential metabolites were screened in group T and group C, of which 38 were upregulated and 43 were downregulated (Figure S2). These differential metabolites mainly included seven categories, namely organic acids and derivatives, lipids and lipid molecules, heterocyclic compounds, benzenoids, organic oxygen compounds, nucleosides and analogues, and hydrocarbon derivatives (Figure 6B).

To more intuitively show the variation in metabolites between the different samples, a hierarchical cluster analysis (HCA) was performed for the top twenty differential metabolite contents in group C and group T (Figure 6C). The columns represent samples, and the rows represent metabolite names. The samples from group T and group C were, respectively, clustered into one class, indicating that the samples in these groups had good repeatability. The contents of differential metabolites between the two groups were relatively different, suggesting that there were great differences between them. The relative content is shown by the difference in color. The red color in the heat map indicates a higher metabolite content, and the blue color indicates a lower metabolite content. The bottom-right region indicates that the metabolite abundance of the group T samples was higher than that of group C, such as L-methionine, S-oxide, L-cystathionine, GMP, etc. The upper left area indicates that the metabolite abundance of the group T samples was lower than that of group C, such as mannose, citric acid, citrulline, etc.

#### 3.5.3. KEGG Enrichment Analysis of Differential Metabolites

**Figure 7:**
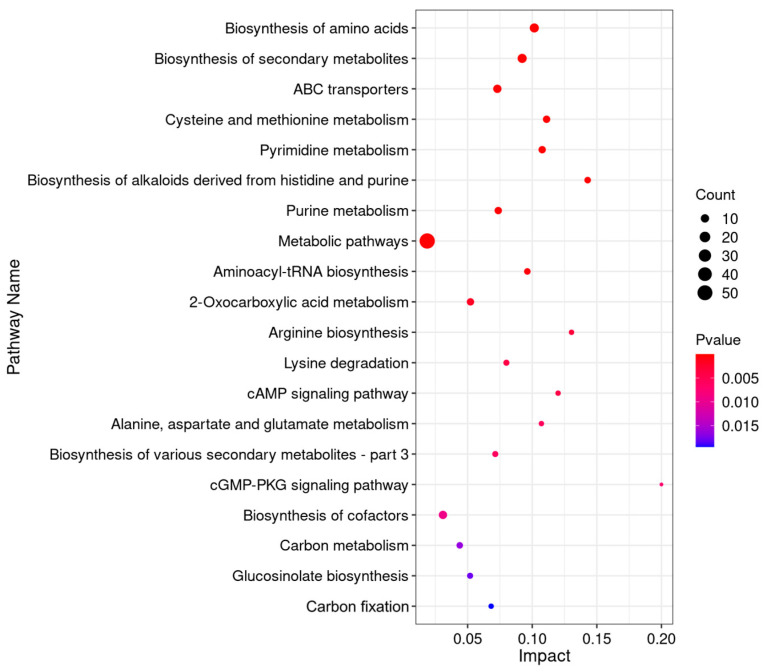
KEGG enrichment analysis of differential metabolites.

KEGG is a powerful tool for metabolism analysis and metabolic network research in vivo. Figure 7 shows the top 20 enrichment pathways with the most significant influence of differentiated metabolites in the comparison between group C and group T. The top five metabolic pathways with the most differential metabolites were the biosynthesis of amino acids, the biosynthesis of secondary metabolites, ABC transporters, cysteine and methionine metabolism, and pyrimidine metabolism.

#### 3.5.4. Differential Metabolite Network Analysis

**Figure 8:**
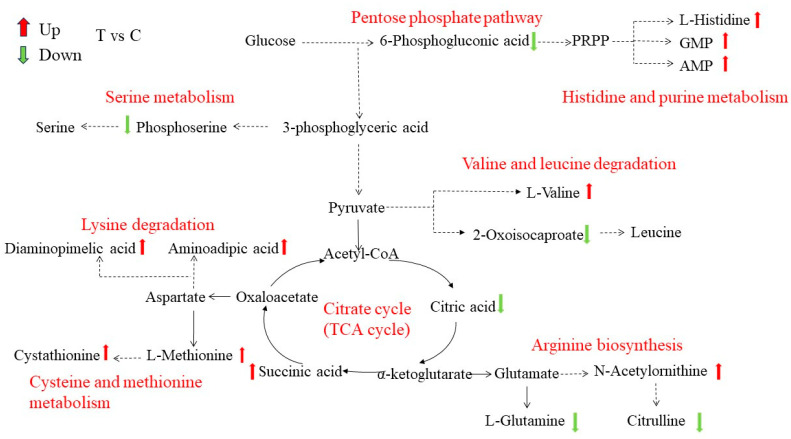
Main differential metabolite metabolic network pathways. Green arrows represent downregulated metabolites, and red arrows represent upregulated differential metabolites. The black fonts represent metabolites, and the red fonts represent metabolic pathways.

According to the different metabolites and KEGG enrichment pathways, a schematic metabolic map was constructed to show the metabolite variation among the two groups, and the changes in each differential metabolite are represented by arrows of different colors (Figure 8). The main differential metabolic pathways were related to carbohydrate metabolism, amino acid synthesis, and energy metabolism. The differential metabolites involved in amino acid synthesis and metabolic pathways mainly include upregulated and downregulated metabolites. The upregulated metabolites include methionine, histidine, valine, cystathionine, etc.; the downregulated metabolites include glutamine, citrulline, citric acid, phosphoserine, 6-phosphogluconic acid, etc. These metabolites were mainly involved in the following physiological metabolisms and processes: the pentose phosphate pathway, histidine and purine biosynthesis, cysteine and methionine metabolism, serine metabolism, valine and leucine degradation, arginine biosynthesis, lysine degradation, the TCA cycle, etc.

## 4. Discussion

*A. alternata*, as a common postharvest pathogenic fungus, can infect fruits and vegetables and cause black spots, leading to food safety problems and huge economic losses [26]. In recent years, many natural antibacterial substances have been found to effectively inhibit the growth of *A. alternata*, such as ferulic acid, γ-aminobutyric acid, methyl jasmonate, natamycin, etc. [27,28]. Met is an essential amino acid, playing a significant role in response to environmental stress and plant disease resistance [29].

The antifungal experiment in vitro results showed that the Met treatment had a certain inhibitory effect on the mycelial growth and spore germination of *A. alternata*. The inhibitory effect was positively correlated with Met concentration in a low concentration range (≤50 mmolL^−1^), while the inhibitory effect decreased when the Met concentration was higher than 50 mmolL^−1^. This result was similar to that previously reported by Liu et al. [30]. The cell membrane, which is crucial for maintaining the normal physiological metabolism of pathogenic fungi, is an important structural component of microorganisms. Meanwhile, it is also the main target of environmental stress damage and the sensor of stress stimulation [31]. The results showed that after the Met treatment, the change rate of electrolyte leakage increased, and the leakage of nucleic acids and proteins increased as well. PI is a fluorescent dye of cell membranes, releasing red fluorescence through penetrating damaged cell membranes and binding to nucleic acids in a cell. The more severely damaged the cell membrane, the redder the color [32]. Compared with the control group, red fluorescence in the treated groups with different concentrations of Met treatment appeared, indicating that the integrity of the cell membranes was damaged. Moreover, the group treated with 50 mmolL^−1^ of Met treatment had the reddest color, which proved that damage to the integrity of the cell membranes was the cause of cell leakage and increased electrolyte leakage. The fungal cell wall, which is an important barrier in maintaining cell morphology, protects cells from adverse factors [33]. The SEM results showed that the Met treatment made the surface of the mycelium of *A. alternata* exhibit obvious distortion, depression and shrinkage. This further proved that Met can destroy the cell wall and membrane of *A. alternata*. Therefore, we speculated that Met treatment might inhibit mycelial growth and spore germination by destroying the integrity of the cell wall and membrane of *A. alternata*, resulting in the leakage of cell contents (electrolytes, nucleic acid, and proteins). This was similar to the antifungal mechanisms of ε-polylysine and γ-aminobutyric acid on *A. alternata* [34].

**Figure 9:**
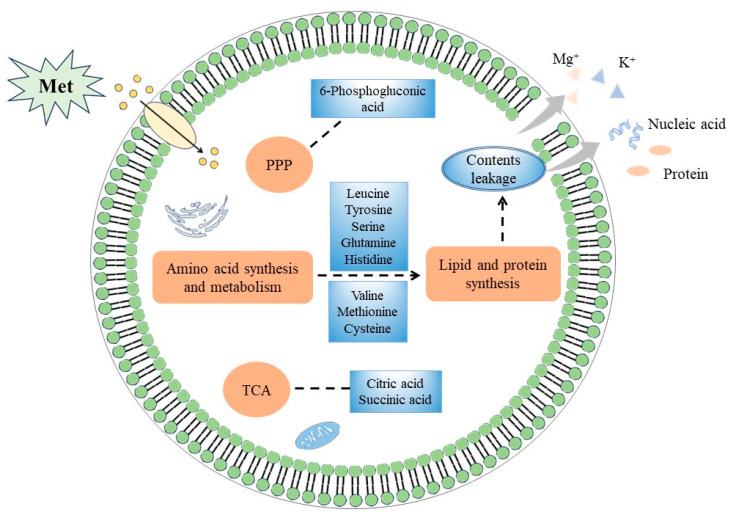
Possible inhibitory mechanism of Met treatment on *A. alternata*.

Sugars, proteins, and lipids are essential substances for microbial growth. In an adverse environment, the synthetic pathway of these substances may be affected, and their contents can serve as an indicator of the metabolic status of cells. As shown in Figure 9, the metabolome analysis showed that there were significant differences in intracellular metabolite levels between the Met-treated group and the control group, such as lipid and lipid molecules, amino acids, organic acids, and derivatives. The pentose phosphate pathway (PPP) is the main pathway of carbohydrate metabolism, providing NADPH for the synthesis of various substances in cells and precursors for the synthesis of amino acids and nucleotides [35]. The metabolome analysis showed that the level of 6-phosphogluconic acid involved in the PPP was downregulated after the Met treatment. Kim et al. proved that interference with the PPP might affect energy and nucleotide output and have a direct impact on some important functions, such as biofilm formation and resistance to stressors [36]. The TCA cycle, which is an important pathway of energy metabolism, produces a variety of intermediate products needed by microorganisms. After the Met treatment, the level of citric acid in the TCA cycle was downregulated, and the level of succinic acid was upregulated. Citric acid and succinic acid were involved in cytoplasmic metabolism and energy metabolism in the mitochondria, so these metabolites were disrupted [37]. In addition, the metabolites of the TCA cycle were required for pyrimidine and lipid synthesis. For example, oxaloacetic acid and citric acid produced cytoplasmic aspartic acid and acetyl CoA, respectively [38]. Therefore, it could be inferred that Met treatment might affect the energy metabolism and carbohydrate metabolism of *A. alternata* by regulating the PPP and TCA cycles.

The metabolome analysis also showed that the Met treatment interfered with various amino acid metabolisms in *A. alternata*. Leucine, arginine, glutamine, serine, and other metabolites were downregulated, while histidine was upregulated. Leucine, which is involved in lipid metabolism, is a component of cell membranes and plays an important role in maintaining the integrity of cell membranes [39,40]. Arginine and glutamine are important sources of nitrogen in cells. Arginine participates in protein synthesis, immune response, urea cycle, and other biological processes. Glutamine can be hydrolyzed to glutamate, playing an important role in cell protection by enhancing the stability of cell membranes [41,42,43]. Serine plays an important role in cell membrane production and the maintenance of intracellular homeostasis [44]. Histidine biosynthesis, which plays an important role in cell metabolism, is associated with purine synthesis and nitrogen metabolism, known as the crossroads of metabolism. However, histidine, as a nitrogen source, does not support cell growth, reduces the growth rate, delays the lag stage, and inhibits the growth of strains. In addition, due to the unique imidazole ring of histidine’s side chain, it has a high affinity for many compounds. This characteristic affects cell adhesion and growth, leading to the failure of biofilm formation [45,46]. Meanwhile, the Met treatment promoted the cysteine, methionine, and valine metabolisms of *A. alternata*. Valine is involved in protein synthesis, and methionine and cysteine stabilize the conformation of proteins by forming disulfide bonds. It might be that the stimulation of foreign Met enhanced the metabolism of certain amino acids to regulate cell homeostasis, which is similar to the results reported by Li et al. [47]. The change in these amino acid contents caused disruptions to cell membrane formation and stability, inhibiting the growth of *A. alternata*.

In short, the Met treatment affected amino acid synthesis and metabolism, the PPP, and the TCA cycle in *A. alternata*, thus interfering with intracellular metabolism and causing serious damage to cells. Meanwhile, the integrity of the cell membrane was also damaged, which caused the cell contents to leak; the activity of *A. alternata* decreased; and growth and development were also inhibited.

## 5. Conclusions

After the Met treatment, the mycelial growth and spore germination of *A. alternata* were inhibited, and the cell membrane permeability and content leakage increased. The metabolomics analysis showed that metabolites such as citric acid and glutamine were downregulated, while metabolites such as histidine and methionine were upregulated. These metabolites, involved in amino acid metabolism, the pentose phosphate pathway, and the TCA cycle in *A. alternata*, affected energy metabolism and membrane integrity and resulted in the leakage of cell contents, thereby inhibiting the growth of *A. alternata*. The results of this study provide a basis for exploring the antifungal mechanism of Met, suggesting that Met could be used as an alternative drug to inhibit *A. alternata* in postharvest fruits and vegetables.

## Figures and Tables

**Table 1:** Effect of different concentrations of Met on colony diameter of *A. alternata*.

Time (h)	Control	5 mmolL^−1^	25 mmolL^−1^	50 mmolL^−1^	100 mmolL^−1^
24	17.73 ± 0.251 ^a^	16.50 ± 0.500 ^b^	16.43 ± 0.513 ^b^	15.86 ± 0.230 ^bc^	15.40 ± 0.529 ^c^
48	32.60 ± 0.360 ^a^	28.36 ± 0.321 ^b^	27.66 ± 0.577 ^bc^	26.56 ± 0.513 ^c^	27.90 ± 1.014 ^b^
72	47.26 ± 0.250 ^a^	43.60 ± 0.529 ^b^	41.26 ± 1.101 ^c^	40.66 ± 1.258 ^c^	42.26 ± 0.873 ^bc^
96	62.29 ± 0.611 ^a^	55.33 ± 0.577 ^b^	53.90 ± 1.014 ^b^	51.23 ± 0.680 ^c^	53.66 ± 1.154 ^b^
120	74.46 ± 0.503 ^a^	66.06 ± 0.404 ^b^	61.73 ± 1.750 ^c^	59.66 ± 0.577 ^d^	60.50 ± 0.500 ^cd^

Note: data are means ± SD (*n* = 3). Columns with different letters at each time point indicate significant differences (*p* < 0.05).

## Data Availability

The data supporting the conclusions of this study are available and included within the article.

## Supplementary Materials

The following supporting information can be downloaded at: https://www.mdpi.com/article/10.3390/jof10020151/s1, Table S1: Primary differential metabolite screening; Figure S1: Histogram of primary differential metabolites.; Table S2: Secondary differential metabolite screening; Figure S2: Histogram of secondary differential metabolites.
