# Supplementary material for: Inhibitory Effect of L-Methionine on Alternaria alternata Based on Metabolomics Analysis
Source: J Fungi (Basel). 2024 Feb 13;10(2):151. doi: 10.3390/jof10020151 (PMC10890048; doi:10.3390/jof10020151)

Supplementary Tables and Figures

Table S1 Primary differential metabolite screening

Groups	TvsC
Total metabolite count	10556
Total differential metabolite count	1916
Up-regulated differential metabolite number	930
Down-regulated differential metabolite numbers	986

Figure S1: Histogram of primary differential metabolites.

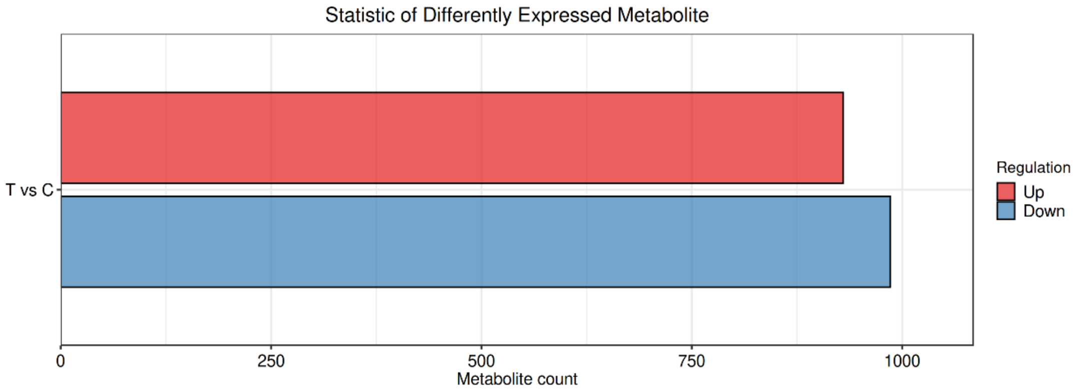

Table S2 Secondary differential metabolite screening

Groups	TvsC
Total metabolite count	332
Total differential metabolite count	81
Up-regulated differential metabolite number	38
Down-regulated differential metabolite numbers	43

Figure S2: Histogram of secondary differential metabolites.

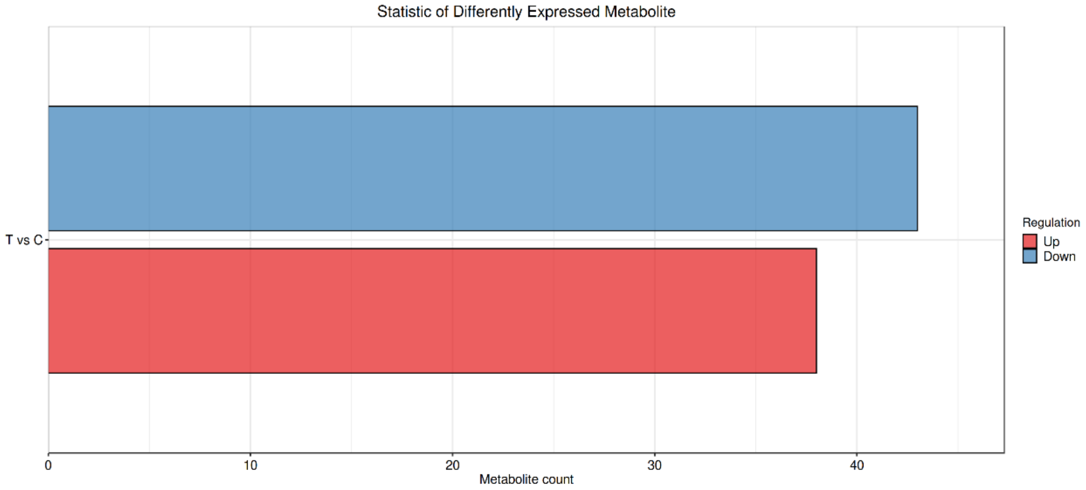

Supplement: Supplementary file 1 [file jof-10-00151-s001.zip › jof-2845803-supplementary.pdf]
